# Prognostic biomarkers in ischemic stroke treated with mechanical thrombectomy: a systematic review

**DOI:** 10.1055/s-0045-1812301

**Published:** 2025-10-31

**Authors:** Rodrigo Fellipe Rodrigues, Raquel Cristina Trovo Hidalgo, Savio Batista, Júlia Belone Lopes, Gabriel Paulo Mantovani, Pedro Henrique Matos Oliveira, André Nishizima, Anderson Silva Corin, Lucas Macedo, Mariana Letícia de Bastos Maximiano, Pedro Lucas Machado Magalhães, Julia Camargo Ricci, Sonia Maria Oliani

**Affiliations:** 1 Universidade Federal de São Paulo, Programa de Pós-Graduação em Biologia Estrutural e Funcional, São Paulo, SP, Brazil.; 2 University of Toronto, Department of Neurosurgery, Division of Interventional Neuroradiology, St. Michael's Hospital, Toronto, ON, Canada.; 3 Faculdade de Medicina de São José do Rio Preto, Departamento de Neurorradiologia Intervencionista, São José do Rio Preto, SP, Brazil.; 4 Emory University, Department of Neurology, Atlanta, GA, USA.; 5 Faculdade de Medicina de São José do Rio Preto, Departamento de Oncologia Clínica, São José do Rio Preto, SP, Brazil.; 6 University of Toronto, Department of Medical Oncology, Sunnybrook Health Science Centre, Toronto, ON, Canada.; 7 Hospital de Clínicas de Porto Alegre, Departamento de Neurologia, Porto Alegre, RS, Brazil.; 8 Escola Bahiana de Medicina e Saúde Pública, Departamento de Medicina, Salvador, BA, Brazil.; 9 Universidade Federal de Pelotas, Departamento de Medicina, Pelotas, RS, Brazil.; 10 Universidade de Brasília, Departamento de Medicina, Brasília, DF, Brazil.; 11 Universidade Federal Fluminense, Departamento de Medicina, Rio de Janeiro, RJ, Brazil.; 12 Instituto de Educação Médica, Departamento de Medicina, Angra dos Reis, RJ, Brazil.; 13 Universidade Estadual Paulista "Júlio de Mesquita Filho", Instituto de Biociências, Letras e Ciências Exatas, São José do Rio Preto, SP, Brazil.

**Keywords:** Ischemic Stroke, Thrombectomy, Biomarkers, Treatment Outcome

## Abstract

**Background:**

Mechanical thrombectomy (MT) is a key therapy for acute ischemic stroke (AIS), improving survival and functional outcomes. However, the variability in results highlights the need for predictive markers to refine patient selection. Biomarkers reflecting inflammation and metabolic stress are gaining recognition for their role in AIS and MT outcomes.

**Objective:**

To systematically review and synthesize the evidence on biomarkers associated with clinical outcomes in AIS patients undergoing MT. Specific aims include evaluating their relationship with functional recovery (mRS), mortality, infarct volume, hemorrhagic transformation, and complications such as malignant brain edema (MBE) and delayed cerebral ischemia (DCI).

**Methods:**

A systematic review of the literature was conducted in accordance with the guidelines of the Preferred Reporting Items for Systematic Reviews and Meta-Analyses (PRISMA) statement to identify studies evaluating biomarkers in MT. The PubMed and Embase databases were searched using the following terms: ( *Marker* OR *biomarker* *) AND ( *Mechanical Thrombectomy* OR *endovascular* ) AND *Stroke* .

**Results:**

Of 2,834 articles identified, 86 met inclusion criteria. Several biomarkers, such as C-reactive protein (CRP), neutrophil-to-lymphocyte ratio (NLR), adenosine deaminase (ADA), neuron-specific enolase (NSE), and matrix metalloproteinase-9 (MMP-9), were consistently associated with worse functional outcomes, increased mortality, and higher risk of complications including hemorrhagic transformation and MBE.

**Conclusion:**

Multiple biomarkers demonstrate prognostic value in AIS patients undergoing MT. These findings may support risk stratification and individualized care, though further prospective studies are needed to integrate these biomarkers into the clinical practice.

## INTRODUCTION

Mechanical thrombectomy (MT) has emerged as the cornerstone treatment for acute ischemic stroke (AIS) caused by large vessel occlusion (LVO), significantly improving clinical outcomes and survival rates in eligible patients. 1 While advancements in MT technology have led to higher recanalization rates, not all patients benefit equally from the procedure. A significant portion of them suffer from poor neurological outcomes despite successful recanalization, a phenomenon known as *futile recanalization* . 2 This discrepancy underscores the need for additional predictive markers that can aid in patient selection, procedural planning, and postoperative management.

Biomarkers, which provide biological insights into a patient's physiological state, have gained increasing attention in the context of stroke and MT. 3 They can offer critical information on underlying mechanisms such as inflammation, metabolic stress, endothelial damage, and coagulation imbalance, all of which are key factors influencing stroke outcomes. 4 Identifying reliable biomarkers could enhance the ability to predict which patients are more likely to experience favorable outcomes and which are at higher risk of complications such as malignant brain edema (MBE) or delayed cerebral ischemia (DCI).

In the current review, we aimed to systematically synthesize current evidence on the prognostic value of biomarkers in patients undergoing mechanical thrombectomy for AIS. We specifically focused on their associations with clinically relevant outcomes, including functional recovery, mortality, infarct volume, and postprocedural complications, such MBE and DCI.

## METHODS

A systematic review of the literature was conducted in accordance with the guidelines of the Preferred Reporting Items for Systematic Reviews and Meta-Analyses (PRISMA) statement to identify studies evaluating biomarkers in MT. The PubMed and Embase databases were searched using the following terms: ( *Marker* OR *biomarker* *) AND ( *Mechanical Thrombectomy* OR *endovascular* ) AND *Stroke* .

**Figure 1:**
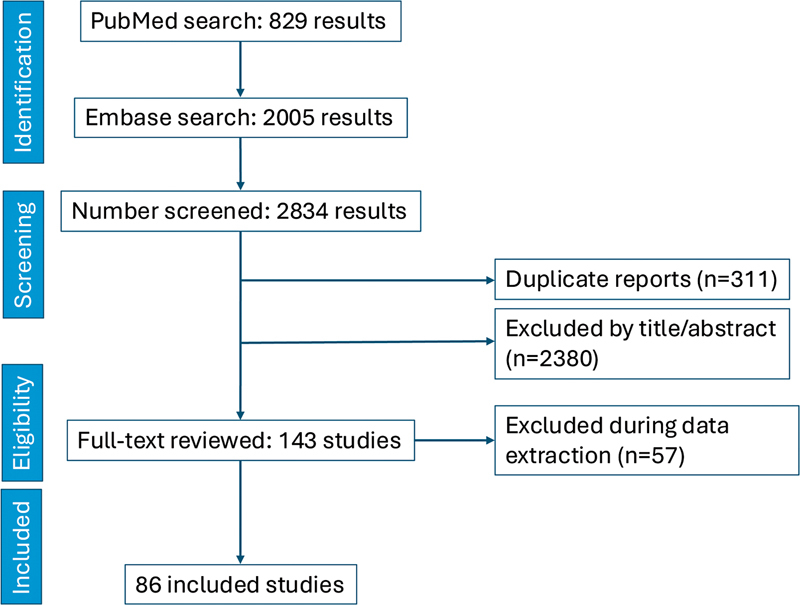
PRISMA flow diagram of study screening and selection process.

The inclusion criterion was clinical studies on the predictive value of biomarkers in patients undergoing MT for strokes. Studies were included if they assessed biomarkers related to outcomes after MT. Nonhuman studies, review articles, and commentaries were excluded. Data were extracted on study design, patient population, type of biomarker, and the outcomes measured, including successful reperfusion, mortality, neurological recovery, and complications such as MBE and DCI. The study selection process is summarized in a PRISMA flow diagram ( Figure 1 ).

## RESULTS

The initial search yielded 2,834 articles, with 311 duplicates removed. Titles and abstracts were screened for relevance, and 143 articles were selected for full-text review. After applying exclusion criteria, 86 articles were included in the final analysis. Table 1 summarizes the outcomes found regarding these biomarkers.

**Table 1:** Predictive outcomes of biomarkers in post-stroke recovery following mechanical thrombectomy

Biomarker	Predictive Outcome
ADA 5	NIHSS and mRS on discharge
CCR4 32	Infarct volume, edema volume
N-acetylmuramoyl-L-alanine amidase 33	Intracranial hemorrhage post-rtPA
Β-Synuclein	ASPECTS, mRS
Adiponectin 33	Stroke risk, mortality
SIPI 6	90-day mRS (3–6)
Syndecan-1 30 31	Survival, NIHSS
SUA/SCr Ratio 34 35	mRS (> 2), stroke severity (NIHSS)
GAR 36 37 38	All-cause mortality
PTFV1 35	END
CRP 3 6 7 8 9 10	Mortality, hospital stay, MBE
NLR 11 12 13 14 15 16 17 18 19 20 21 22 23 24 25 26 27 28 29	Revascularization failure, mortality, poor functional outcome
PAL 39	MBE, mortality (cutoff: 1.6 mmol/L)
NPR 17	Futile recanalization, hospital mortality
WBC and RBCs 3 7 17	Inflammation, metabolic state; RDW: mRS, all-cause mortality
MPV 7	mRS
vWF 23	Thrombogenesis, DCI
NETs and H3Cit 23 24	Vascular damage, inflammation
Interleukins (IL-6, IL-8, IL-10) 7 8 9	DCI, mortality, MBE
MCP-1 and sTNFRI 9	Poor recovery, systemic inflammation
Soluble P-selectin and VCAM-1 9	DCI, 90-day mRS
MMP-9 10	Hemorrhagic transformation, poor functional recovery
BD-Tau, NfL, GFAP 28	Poor 90-day outcome
Long-chain ceramides, Sphingosine-1-phosphate 29	Mortality, recovery
Prealbumin, S100B, NSE 30 31	Blood-brain barrier disruption, poor outcome
D-Dimer, FAR 6 8	Mortality, coagulation

Abbreviations: ADA, adenosine deaminase; ASPECTS, Alberta Stroke Programme Early CT Score; BD-Tau, brain-derived tau; CRP, C-reactive protein; DCI, delayed cerebral ischemia; END, early neurological deterioration; FAR, fibrinogen to albumin ratio; GAR, glucose-to-glycated hemoglobin ratio; MBE, malignant brain edema; MCP-1, monocyte chemoattractant protein-1; MMP-9, matrix metalloproteinase-9; MPV, mean platelet volume; mRS, modified Rankin Scale; NETs, neutrophil extracellular traps; NIHSS, National Institutes of Health Stroke Scale; NLR, neutrophil-to-lymphocyte ratio; NPR, neutrophil-to-olatelet ratio; NSE, neuron-specific enolase; PAL, peripheral arterial lactate; PTFV1, P-wave terminal force in lead V1; RBCs, red blood cells; rtPA, recombinant tissue plasminogen activator; SIPI, systemic inflammatory protein index; SUA/SCr, serum uric acid-to-serum creatinine ratio; vWF, von Willebrand factor; WBC, white blood cells.

### Adenosine deaminase

The immune response enzyme adenosine deaminase (ADA) was significantly associated with higher scores on the National Institutes of Health Stroke Scale (NIHSS) and poorer modified Rankin Scale (mRS) scores at discharge, indicating greater neurological impairment and limited functional recovery. Elevated levels suggest heightened systemic inflammation, which contributes to adverse outcomes, making ADA a critical inflammatory predictor in poststroke recovery. 5

### Systemic inflammatory protein index

The systemic inflammatory protein index (SIPI), reflecting systemic inflammation, showed strong correlation with unfavorable outcomes, defined by a 90-day mRS of 3 to 6. Higher scores were indicative of prolonged recovery periods and reduced functional independence, emphasizing the role of systemic inflammatory responses in determining long-term disability. 6

### C-reactive protein

As a well-established inflammation marker, C-reactive protein (CRP) levels above 20.5 mg/L correlated with poor neurological outcomes, prolonged hospital stays, and MBE ( *p* < 0.05). Its elevation aligns it closely with ADA and SIPI as a predictor of systemic inflammation, suggesting that heightened CRP levels increase the risk of adverse clinical outcomes. 3 6 7

### Neutrophil-to-lymphocyte ratio

As a robust marker of immune activation, the neutrophil-to-lymphocyte ratio (NLR) was associated with failed revascularization, increased mortality, and poor functional outcomes. 34 Elevated NLR levels reflect an intense immune response to ischemic insult, positioning it as a valuable inflammatory marker that parallels ADA and CRP in predicting recovery challenges. 8 9 10 11

### Syndecan-1

The endothelial marker Syndecan-1 was elevated in non-survivors and patients with neurological deterioration, with significant declines postendovascular therapy ( *p* < 0.001). Higher baseline levels indicate greater endothelial damage, correlating with worse survival outcomes. Syndecan-1's role in vascular injury parallels that of other inflammatory markers, such as SIPI and CRP, in predicting recovery limitations. 12 13

### Chemokine receptor type 4 (CCR4)

As an immune cell trafficking receptor, chemokine receptor type 4 (CCR4) was linked with infarct volume and edema. Machine learning models identified it as predictive of ischemic damage, where elevated levels correlate with extensive infarct volume and edema, contributing to structural brain damage and influencing functional outcomes. 14

### N-acetylmuramoyl-L-alanine amidase

Associated with immune response, elevated N-acetylmuramoyl-L-alanine amidase levels were correlated with an increased risk of intracranial hemorrhage (ICH) postrecombinant tissue plasminogen activator (post-rtPA) treatment in stroke patients, underscoring its value in predicting hemorrhagic complications. This marker aids in identifying patients who may be at higher risk of posttreatment hemorrhage. 15

### Serum uric acid-to-serum creatinine ratio

As a metabolic stress indicator, the serum uric acid-to-serum creatinine (SUA/SCr) ratio was associated with poor functional outcomes (mRS > 2) and severe stroke (higher NIHSS scores). Elevated ratios indicate oxidative stress, similar to inflammatory markers like the SIPI, emphasizing its predictive value in assessing recovery needs. 16

### Glucose-to-glycated hemoglobin ratio

The glucose-to-glycated hemoglobin ratio (GAR), a marker of glycemic control, was significantly associated with all-cause mortality. Elevated ratios reflect systemic metabolic dysfunction, with higher levels correlating with increased mortality risk. Its prognostic role in survival outcomes positions it alongside SUA/SCr as a critical metabolic biomarker in poststroke care. 17

### Peripheral arterial lactate

A marker of metabolic stress, peripheral arterial lactate (PAL) levels above 1.6 mmol/L were linked to MBE and increased mortality ( *p* < 0.01). This marker provides insights into anaerobic metabolism during ischemia, reinforcing its predictive role in adverse recovery outcomes and metabolic challenges poststroke. 18

### Β-synuclein

The Β-synuclein is a neurodegeneration-associated protein, which is strongly linked to lower Alberta Stroke Programme Early CT Score (ASPECTS) and higher mRS scores at discharge. Elevated levels indicate extensive ischemic injury and limited recovery, positioning Β-Synuclein as a marker of neurodegenerative damage and prognosis. 19

### White and red blood cells

Higher white blood cell (WBC) counts were correlated with systemic inflammation and post-MT complications, while abnormal red blood cell (RBC) counts reflected underlying pathophysiological disruptions. Both counts offer valuable insights into the patient's inflammatory and metabolic status, influencing recovery outcomes, with red cell distribution width (RDW) specifically linked to mRS and all-cause mortality. 20 21

### Mean platelet volume

There was an association between the mean platelet volume (MPV) and the mRS scores, suggesting that larger platelet size could predict increased stroke severity and poorer functional outcomes, aiding in identifying patients who may require closer monitoring poststroke. 22

### Von Willebrand factor

As an endothelial marker, elevated Von Willebrand factor (vWF) levels were associated with DCI and thrombotic complications, mirroring the role of syndecan-1 in predicting vascular injury. Monitoring vWF levels may aid in guiding treatment strategies, particularly for thrombotic risk after MT. 23

### Neutrophil extracellular traps and H3Cit

Neutrophil extracellular traps (NETs), especially citrullinated histone H3 (H3Cit), are key markers of neutrophil activation linked to thrombus formation. Elevated H3Cit levels correlate with vascular damage and inflammatory outcomes, emphasizing the role of NETs in poor stroke recovery due to enhanced thrombus formation. 23 24

### Interleukins 6, 8, and 10

Elevated levels of interleukin 6 (IL-6), above 10 thousand pg/mL (area under the curve [AUC] = 0.82; *p* < 0.001), correlated with DCI and mortality, with IL-8 associated with MBE and IL-10 with adverse recovery. This cytokine profile reflects both proinflammatory and counter-regulatory responses, adding depth to inflammatory and outcome predictions after stroke. 7 8 25

### Monocyte chemoattractant protein-1 and soluble tumor necrosis factor receptor I

Both Monocyte chemoattractant protein-1 (MCP-1) and soluble tumor necrosis factor receptor I (sTNFRI) were associated with increased risk of poor recovery, indicating heightened inflammatory response. These markers underscore the role of inflammation in stroke outcomes, furthering insights into systemic immune responses. 25 26

### Soluble P-selectin and vascular cell adhesion molecule-1

Elevated levels of soluble P-selectin and vascular cell adhesion molecule-1 (VCAM-1) indicative of endothelial dysfunction, were linked to DCI and poor 90-day mRS outcomes ( *p* < 0.01). Both are essential in assessing endothelial damage post-MT, aiding in predicting secondary complications. 26

### Matrix metalloproteinase-9

As a blood-brain barrier integrity marker, elevated matrix metalloproteinase-9 (MMP-9) levels were associated with hemorrhagic transformation and poor functional recovery, highlighting its role in vascular injury assessment after MT. 10 26 27

### Brain-derived tau, neurofilament light chain, and glial fibrillary acidic protein

These neurodegenerative markers were strongly associated with poor 90-day outcomes. Elevated levels of brain-derived tau (BD-Tau), neurofilament light chain (NfL), and glial fibrillary acidic protein (GFAP) indicated neuronal and astrocytic damage, highlighting them as essential markers for predicting long-term neurological impairment. 28

### Long-chain ceramides and sphingosine-1-phosphate

Elevated levels of ceramides and sphingosine-1-phosphate were correlated with increased mortality, positioning them as metabolic markers reflecting cellular apoptosis and inflammation in ischemic stroke recovery. 29

### Prealbumin, S100B, and neuron-specific enolase

Prealbumin, S100B, and neuron-specific enolase (NSE), associated with neuronal and glial injury, were linked to blood-brain barrier disruption and poorer outcomes. Elevated levels of S100B, in particular, serve as predictors of severe brain injury, providing a measure of injury severity. 30 31

### D-dimer and fibrinogen-to-albumin ratio

The level of D-dimer and the fibrinogen-to-albumin ratio (FAR) were linked to coagulation and thrombolysis, being associated with higher mortality rates, underscoring their role in assessing coagulation status and recovery potential in MT patients. 32

### Adiponectin

Adiponectin has been associated with the severity of stroke and may function as a predictive biomarker for adverse outcomes and mortality after stroke, emphasizing its potential importance in poststroke prognosis. 33

### Neutrophil-to-platelet ratio

A low platelet-to-lymphocyte ratio and high neutrophil-to-platelet ratio (NPR) have been linked to increased risks of post-EVT hemorrhage and futile recanalization in AIS patients with large vessel occlusions. 34

### P-wave terminal force in lead V1

Several electrocardiogram (ECG) indicators representing potential atrial remodeling have been independently associated with stroke and atrial fibrillation (AF), including P-wave duration (PWD), P-wave dispersion, interatrial blocks, P-wave terminal force in lead V1 (PTFV1), and P-wave axis. 35

## DISCUSSION

It is important to note that this systematic review highlights associations between biomarkers and outcomes but does not support direct treatment recommendations or contraindications. Only randomized controlled trials can provide sufficient evidence for clinical decision-making. Observational and systematic studies are hypothesis-generating in nature.

Biomarkers, like the ones mentioned in the current study, have gained considerable attention over recent decades for their potential to improve diagnosis, treatment, and prognostic precision across various diseases, including stroke. In cardiology, biomarkers such as troponin are essential for assessing myocardial injury in acute coronary syndromes and predicting cardiovascular events, underscoring the connection between cerebro- and cardiovascular diseases. Oncology is similarly rich in biomarkers, with some, like NSE and CRP, being critical for cancer diagnosis and prognosis. Both stroke and cancer involve shared mechanisms of inflammation and cellular injury. 36

Over the past decade, biomarker research in neuropsychiatry has expanded significantly, with new ones like amyloid and tau proteins emerging in studies of neurodegenerative and psychiatric conditions. This evolution reflects an increasing understanding of the relationship between brain and mental health, positioning biomarkers as tools for exploring neuroinflammation and neuronal degeneration. 37 38

Biomarker identification has proved beneficial in elucidating physiological and pathological processes and in evaluating clinical and pharmacological responses to treatment. However, challenges remain in neurology, including the complexity of brain diagnosis, limited functional endpoints, and high technical costs. In this field, biomarkers could offer insight across the entire spectrum of disease, from early manifestations to end stages. 39

Limitations of this study include heterogeneity in biomarker measurement techniques, variability in outcome definitions, potential publication bias, and lack of patient-level data for meta-analysis. These factors reduce the ability to draw strong conclusions or establish causality.

In stroke, specific biomarkers offer valuable insights into the disease's underlying mechanisms, such as inflammation, neuronal injury, and oxidative stress, which aid in understanding cerebrovascular pathophysiology. Certain biomarkers correlate with patient outcomes, helping predict recovery potential and long-term disabilities. For instance, elevated S100B protein levels are associated with worse outcomes, while markers like D-dimer, CRP, and NSE assist in distinguishing between ischemic and hemorrhagic strokes, as well as gauging event severity, corroborating studies by Jickling and Kamtchum-Tatuene. Moreover, NSE levels post-MT have shown predictive value for poor outcomes and sICH, aligning with findings by Mechtouff and Wang et al.. 3

The use of biomarkers in MT has gained momentum alongside advances in endovascular stroke therapy. These may optimize patient selection by refining criteria beyond traditional imaging and clinical assessments, potentially improving outcomes. Additionally, biomarkers can monitor treatment responses and predict complications like hemorrhagic transformation, enabling proactive management strategies. Continued research into this topic may reveal specific therapeutic targets to enhance thrombectomy outcomes and guide intervention strategies.

Future research should aim to validate these biomarkers in prospective trials and develop standardized thresholds for clinical application. Some, such as CRP, NLR, and NSE, may be useful for early risk stratification, whereas others like MMP-9 and BD-tau might guide prediction of complications such as hemorrhagic transformation or long-term disability.

In conclusion, this systematic review identified several biomarkers, including CRP, NLR, ADA, S100B, NSE, and MMP-9, that are consistently associated with functional outcomes, mortality, and complications in patients undergoing MT. These biomarkers may improve prognostic assessment and inform postprocedural management. Further prospective studies are needed to establish standardized cutoff values and confirm their utility in guiding clinical decisions.

The identification of reliable biomarkers offers a promising avenue for improving patient outcomes in mechanical thrombectomy. By utilizing them to predict which patients are more likely to experience favorable outcomes or complications such as malignant brain edema, clinicians can tailor treatment approaches to the specific physiological needs of each patient. Moving forward, a more integrated use of biomarkers in clinical practice could further enhance procedural success and patient recovery by allowing for early intervention in high-risk cases.
